# Exogenous Myo-Inositol Promotes Sugar Beet Growth and Nutrient Uptake in Saline-Alkali Soil

**DOI:** 10.3390/plants15071022

**Published:** 2026-03-26

**Authors:** Liyang Wang, Hongrui Xu, Guangyu Ji, Yiao Hu

**Affiliations:** Inner Mongolia Key Laboratory of Soil Quality and Nutrient Resources, Key Laboratory of Agricultural Ecological Security and Green Development at Universities of Inner Mongolia Autonomous Region, College of Resources and Environmental Sciences, Inner Mongolia Agricultural University, Hohhot 010018, China; 15838406476@163.com (H.X.); a2092971936@163.com (G.J.); huyiao1991@163.com (Y.H.)

**Keywords:** sugar beet, myo-inositol, saline-alkali soil, nutrient uptake, ion homeostasis, antioxidant enzymes, root morphology

## Abstract

Saline-alkali stress restricts crop yield by disrupting nutrient and water uptake, ionic balance, and oxidative homeostasis. Although myo-inositol enhances tolerance to abiotic stress, its role in sugar beet (*Beta vulgaris* L.) under saline-alkali conditions remains unclear. To investigate the effects of exogenous myo-inositol on sugar beet growth under saline-alkali soils, a pot experiment was conducted using six myo-inositol concentrations (0, 0.2, 0.4, 0.6, 0.8, and 1.0 g L^−1^). Myo-inositol significantly influenced plant performance in a concentration-dependent manner. The 0.6 g L^−1^ treatment produced the highest shoot and root fresh and dry weights, nearly doubling shoot biomass compared with the control. Shoot N and P contents increased markedly at 0.6 g L^−1^, while their concentrations remained relatively stable, indicating biomass-driven nutrient accumulation. Myo-inositol reduced Na accumulation while maintaining stable K, Ca, and Mg concentrations, thereby improving ionic balance. Antioxidant capacity was enhanced, with superoxide dismutase and catalase activities significantly elevated. Root total length and surface area increased substantially, whereas specific root length and surface area decreased, suggesting improved root morphological development. Soil alkaline phosphatase activity was also stimulated at higher myo-inositol treatments. Overall, moderate myo-inositol application (with regression analysis indicating an optimum of approximately 0.56 g L^−1^) improved sugar beet growth through enhanced nutrient acquisition, ionic balance, antioxidant capacity, and root development, offering practical insights for its use as a growth regulator in saline-alkali crop production.

## 1. Introduction

Soil salinization represents a major global environmental challenge, posing a serious threat to ecological security and sustainable agricultural yield [1]. Globally, salinized soils occupy approximately 1381 million hectares, accounting for 10.7% of the global land area [2]. In China alone, 36.7 million hectares are affected by salinity, including 9.2 million hectares of arable land (6.6% of total cultivated land), mainly distributed in eastern coastal areas and the arid and semi-arid regions of northwestern China [3]. Salinized farmland is characterized by high concentration of soluble salts and limited nutrient availability, leading to impaired water uptake, osmotic imbalance, excessive accumulation of reactive oxygen species (ROS), and sodium ion (Na^+^) retention caused by osmotic stress, oxidative stress, and ionic toxicity [4,5,6]. These stresses disrupt plant metabolic processes, resulting in reduced crop yield and quality and causing substantial losses to the agricultural economy. Given these challenges, sugar beet (*Beta vulgaris* L.) has emerged as a promising crop owing to its strong tolerance to saline-alkali conditions, with the ability to maintain growth and yield under soil salinity levels of approximately 8–16 dS·m^−1^ [7,8,9]. As the second most important sugar crop after sugarcane, it is widely cultivated in the saline-alkali farmlands of northwestern China [10]. Optimizing sugar beet cultivation in saline-alkali areas and implementing effective soil remediation strategies are vital for enhancing yield and ensuring the sustainable use of saline-alkali land [11].

In this context, the use of salinity-stress alleviators has gained increasing attention as an effective strategy for improving plant tolerance under saline conditions. Myo-inositol is an essential physiological metabolite and an important osmotic regulator in eukaryotic cells [12]. In plants, it is the most abundant inositol isomer and serves as a central precursor in inositol metabolism, contributing to the synthesis of diverse derivatives, including inositol polyphosphates, D-glucuronic acid, phosphatidylinositol phosphates, raffinose family oligosaccharides, and methylated compounds [13]. Under stress conditions, these metabolites play critical roles in signal transduction, membrane and cell wall formation, and phosphate storage, thereby contributing to abiotic stress tolerance [13]. Myo-inositol and its derivatives regulate a wide range of physiological processes, including programmed cell death, defense responses, and salt tolerance, highlighting their broad protective functions in plants [14,15,16]. Exogenous myo-inositol application has been shown to enhance plant resistance to both biotic and abiotic stresses. For example, in maize (*Zea mays* L.), exogenous myo-inositol improves photosynthesis, maintains ROS homeostasis by reducing hydrogen peroxide and superoxide accumulation, enhances osmotic regulation, and promotes nutrient uptake, thereby alleviating salt stress [17]. In sorghum (*Sorghum bicolor* L.), it modulates stomatal aperture, optimizing transpiration and CO_2_ uptake, which enhances photosynthetic performance and salinity tolerance [18]. In wheat (*Triticum aestivum* L.), exogenous myo-inositol mitigates salinity stress by reducing Na^+^ accumulation and the Na^+^/K^+^ ratio while facilitating osmotic adjustment through increased accumulation of soluble sugars and other compatible solutes [19]. Nevertheless, the mechanisms by which exogenous myo-inositol regulates physiological processes and enhances saline-alkali stress tolerance in sugar beet remain to be elucidated.

In addition to its physiological and biochemical benefits, the substance also influences root development. Roots are the organs most directly exposed to salinity, and their structural and functional traits are critical determinants of plant tolerance to salt-alkali stress [20]. Stress-adapted species often develop highly plastic root systems with increased total root length, a greater proportion of fine roots, and expanded root surface area, which enhance root–soil interactions and nutrient acquisition under adverse conditions [21,22]. However, saline-alkali stress can markedly inhibit root growth by impairing cell division and elongation, thereby restricting primary root development and reducing root branching [23]. This limitation is particularly pronounced during the seedling stage, when roots are morphologically immature and stress defense capacity is limited [24,25]. Previous studies have shown that exogenous myo-inositol enhances root development in Chinese cabbage under salt stress, as evidenced by increased root vitality, total root length, and root volume, thereby promoting shoot growth and nutrient uptake [26]. Nevertheless, whether exogenous myo-inositol can similarly enhance root development in sugar beet seedlings remains unclear.

Establishing effective exogenous myo-inositol-based regulatory strategies is therefore critical for sugar beet cultivation in saline-alkali soils, particularly during the highly stress-sensitive seedling stage. Saline-alkali stress during this early developmental phase may cause irreversible physiological damage and ultimately reduce crop yield. Therefore, the present study aimed to investigate the responses of sugar beet seedlings grown in saline soil to exogenous myo-inositol application. The following hypotheses were tested: (1) exogenous myo-inositol promotes growth and nutrient uptake in sugar beet seedlings grown in saline soil, with the presence of an optimal application rate; and (2) growth enhancement is associated with improved ionic homeostasis, strengthened antioxidant defense (including peroxidase, POD; superoxide dismutase, SOD; and catalase, CAT), and optimized root growth. The results will provide a comprehensive understanding of myo-inositol-mediated plant response mechanisms under saline-alkali stress through integrated analyses of sugar beet growth, nutrient accumulation, ionic balance, antioxidant enzyme activities, root morphological traits, and soil enzyme activities.

## 2. Results

### 2.1. Plant Growth

**Figure 1:**
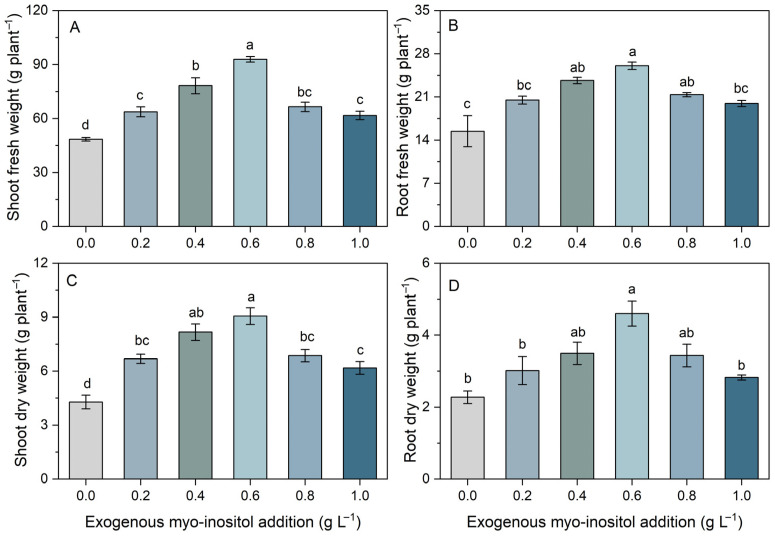
Effects of different concentrations of exogenous myo-inositol (0.0–1.0 g L^−1^) on shoot fresh weight (**A**), root fresh weight (**B**), shoot dry weight (**C**), and root dry weight (**D**) in sugar beet. Values represent means ± SE (*n* = 4). Different letters above the bars indicate significant differences among treatments (*p* ≤ 0.05).

Exogenous myo-inositol application significantly affected plant biomass accumulation, exhibiting a clear dose-dependent response (Figure 1 and Figure S1). Both shoot and root fresh weights increased progressively as myo-inositol concentration rose from 0.0 to 0.6 g L^−1^, followed by a decline at higher concentrations (0.8 and 1.0 g L^−1^). The maximum shoot fresh weight was observed at 0.6 g L^−1^, which was approximately twice that of the 0 g L^−1^ treatment and significantly higher than all other treatments. Similarly, root fresh weight peaked at 0.6 g L^−1^, representing a significant increase compared with the control (approximately 69%) and the 0.2 (27%) and 1.0 g L^−1^ (31%) treatments (Figure 1A,B).

Dry biomass exhibited a comparable pattern (Figure 1C,D), with both shoot and root dry weights peaking at 0.6 g L^−1^ and increasing by 111% and 102%, respectively, compared with the control. Although biomass remained relatively high at 0.8 g L^−1^, further increasing the concentration to 1.0 g L^−1^ significantly reduced both fresh and dry weights. Collectively, these results demonstrate that moderate supplementation with myo-inositol (with regression analysis indicating an optimum of approximately 0.56 g L^−1^, Figure S2) optimally promotes shoot and root biomass accumulation, whereas excessive concentrations exert inhibitory effects on plant growth.

### 2.2. Shoot Nitrogen and Phosphorus Accumulation

**Figure 2:**
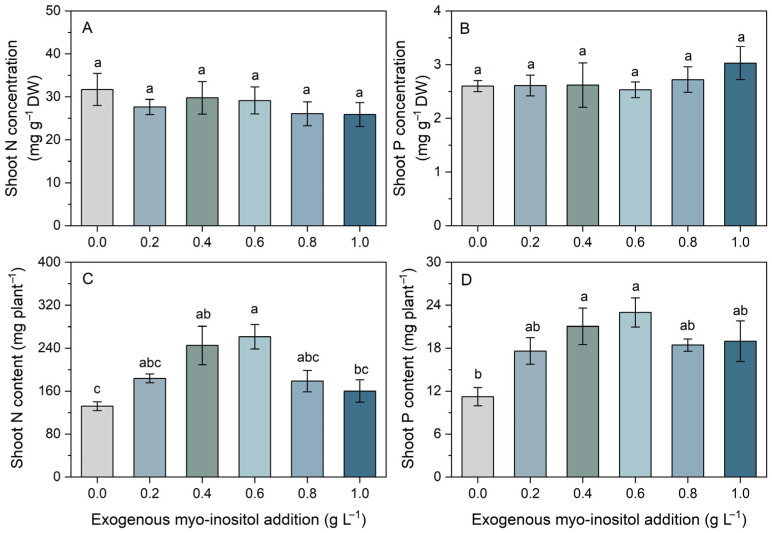
Effects of different concentrations of exogenous myo-inositol (0.0–1.0 g L^−1^) on shoot nitrogen (N) concentration (**A**), shoot phosphorus (P) concentration (**B**), shoot N content (**C**), and shoot P content (**D**) in sugar beet. Values represent means ± SE (*n* = 4). Different letters above the bars indicate significant differences among treatments (*p* ≤ 0.05).

Exogenous myo-inositol significantly influenced shoot N and P contents, whereas nutrient concentrations remained largely unaffected (Figure 2). Shoot N and P concentrations were relatively stable across treatments (0.0–1.0 g L^−1^), with no significant differences detected (Figure 2A,B). In contrast, shoot N and P contents responded markedly to increasing myo-inositol levels. Shoot N content increased progressively from the control to 0.6 g L^−1^ (rising by 98%), reaching its maximum at this concentration, and then decreased at 0.8 and 1.0 g L^−1^ (Figure 2C). A similar pattern was observed for shoot P content, which increased significantly with myo-inositol addition, peaked at 0.6 g L^−1^ (representing a 105% increase relative to the control), and slightly declined at higher concentrations (Figure 2D). Overall, while myo-inositol did not significantly alter shoot N and P concentrations, it substantially enhanced total nutrient accumulation per plant, particularly at 0.6 g L^−1^. These results suggest that enhanced nutrient uptake was primarily driven by increased biomass production rather than changes in tissue nutrient concentration.

### 2.3. Shoot Mineral Ion Concentrations

**Figure 3:**
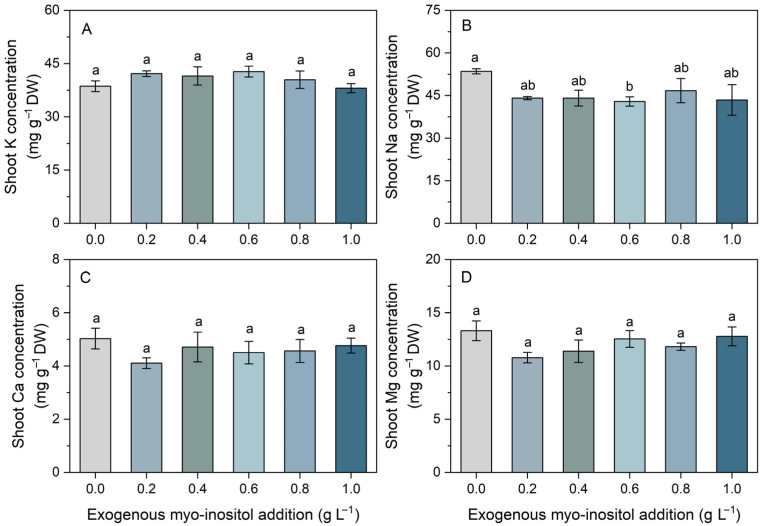
Effects of different concentrations of exogenous myo-inositol (0.0–1.0 g L^−1^) on shoot potassium (K) concentration (**A**), sodium (Na) concentration (**B**), calcium (Ca) concentration (**C**), and magnesium (Mg) concentration (**D**) in sugar beet. Values represent means ± SE (*n* = 4). Different letters above the bars indicate significant differences among treatments (*p* ≤ 0.05).

Exogenous myo-inositol exerted differential effects on shoot mineral ion concentrations (Figure 3). Shoot K concentrations remained stable across all treatments, with no significant differences detected (Figure 3A). Similarly, Ca and Mg concentrations exhibited only minor fluctuations and did not differ significantly among treatments (Figure 3C,D). In contrast, shoot Na concentration showed greater variability. Na levels tended to decrease at 0.2–0.6 g L^−1^ compared with the control, reaching the lowest value at 0.6 g L^−1^. A significant reduction was observed only at 0.6 g L^−1^ relative to the control. At higher concentrations (0.8 and 1.0 g L^−1^), Na levels partially recovered (Figure 3B). Overall, myo-inositol had limited effects on essential cation concentrations (K, Ca, Mg) but moderately reduced Na accumulation at specific concentrations, particularly at 0.6 g L^−1^. This suggests that myo-inositol may enhance ionic homeostasis primarily through selective regulation of Na rather than through alterations in major essential cations.

### 2.4. Antioxidant Enzyme Activities and Malondialdehyde Content

**Figure 4:**
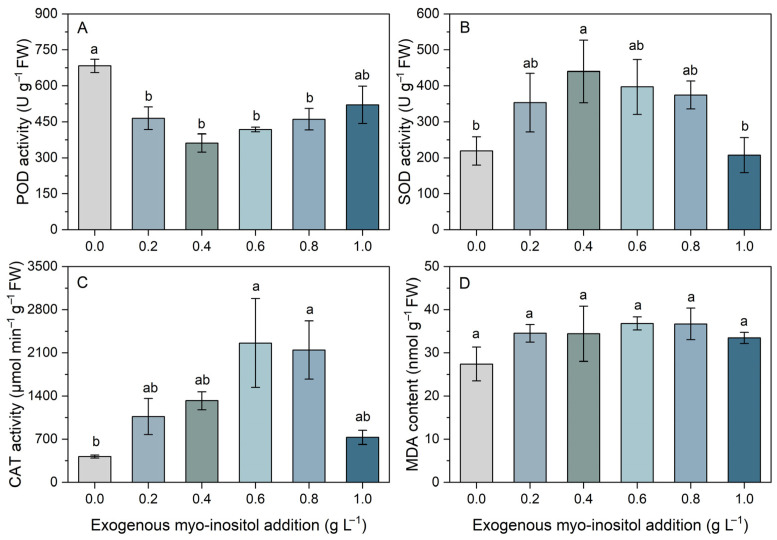
Effects of different concentrations of exogenous myo-inositol (0.0–1.0 g L^−1^) on leaf peroxidase (POD) activity (**A**), superoxide dismutase (SOD) activity (**B**), catalase (CAT) activity (**C**), and malondialdehyde (MDA) concentration (**D**) in sugar beet. Values represent means ± SE (*n* = 4). Different letters above the bars indicate significant differences among treatments (*p* ≤ 0.05).

Myo-inositol application significantly modulated antioxidant enzyme activities (Figure 4). Peroxidase activity was highest in the control and decreased significantly following myo-inositol treatment, reaching its minimum at 0.4 g L^−1^ (approximately 47% lower). Although POD activity partially recovered at higher concentrations (0.8 and 1.0 g L^−1^), it remained lower than the control in most treatments (Figure 4A). Superoxide dismutase activity exhibited a distinct pattern, increasing with myo-inositol concentration up to 0.4 g L^−1^ (approximately 101% higher than the control), where it reached its peak, followed by a decline at 0.6–1.0 g L^−1^ (Figure 4B). Catalase activity increased markedly with increasing myo-inositol concentration and peaked at 0.6 g L^−1^ (over fivefold higher than the control), after which it moderately declined. Notably, CAT activity at 0.6 and 0.8 g L^−1^ was significantly higher than the control (Figure 4C). Malondialdehyde MDA content remained stable at approximately 30 nmol g^−1^ FW across all myo-inositol treatments, with no significant differences among treatments (Figure 4D). Collectively, myo-inositol modulated the antioxidant defense system in a concentration-dependent manner, enhancing SOD and CAT activities at moderate concentrations while reducing POD activity.

### 2.5. Root Morphological Traits

**Figure 5:**
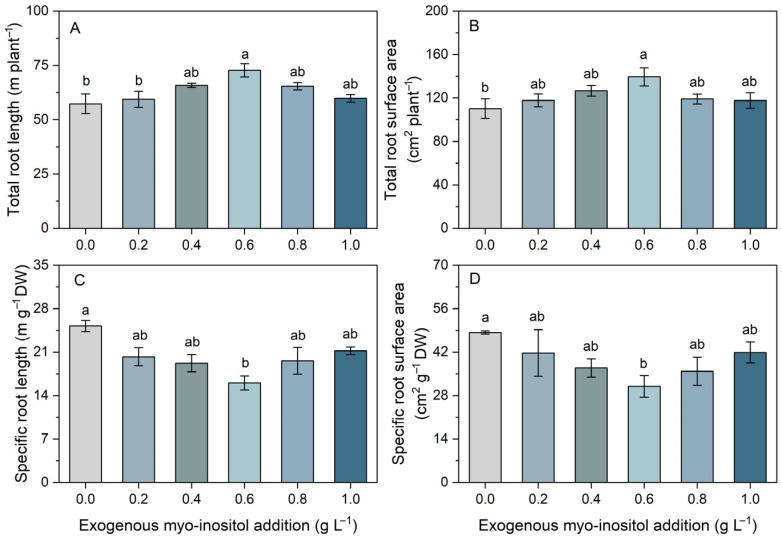
Effects of different concentrations of exogenous myo-inositol (0.0–1.0 g L^−1^) on total root length (**A**), total root surface area (**B**), specific root length (**C**), and specific root surface area (**D**) in sugar beet. Values represent means ± SE (*n* = 4). Different letters above the bars indicate significant differences among treatments (*p* ≤ 0.05).

Myo-inositol significantly influenced root morphological traits in a concentration-dependent manner (Figure 5). Total root length increased progressively up to 0.6 g L^−1^, where it reached a maximum, approximately 27% greater than the control, followed by a slight decline at higher concentrations (Figure 5A). Total root surface area showed a similar pattern, reaching its maximum at 0.6 g L^−1^, with an approximately 26% increase relative to the control (Figure 5B). In contrast, specific root length and specific root surface area displayed the opposite trend. Both parameters were highest in the control and decreased significantly at moderate myo-inositol concentrations, reaching their lowest values at 0.6 g L^−1^ (approximately 36% below the control), with partial recovery at higher concentrations (Figure 5C,D). These results suggest that moderate myo-inositol supplementation enhances root system expansion primarily through increased root biomass and structural development rather than through proliferation of finer roots.

### 2.6. Soil Enzyme Activities and Available Nutrients

**Figure 6:**
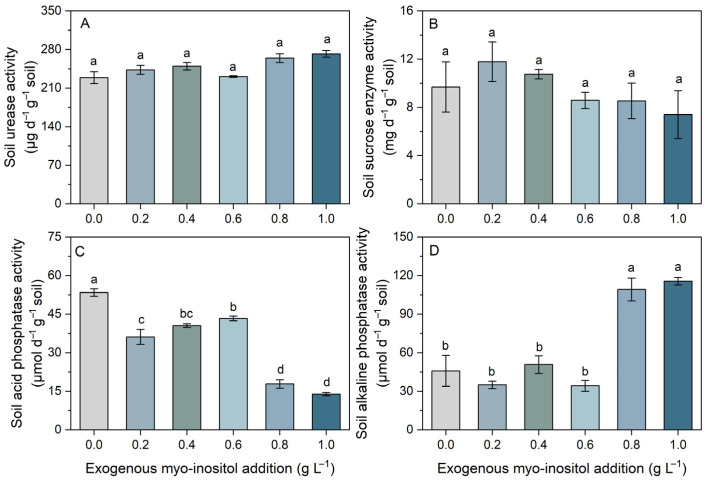
Effects of different concentrations of exogenous myo-inositol (0.0–1.0 g L^−1^) on soil urease activity (**A**), soil sucrase activity (**B**), soil acid phosphatase activity (**C**), and soil alkaline phosphatase activity (**D**). Values represent means ± SE (*n* = 4). Different letters above the bars indicate significant differences among treatments (*p* ≤ 0.05).

Myo-inositol differentially affected soil enzyme activities (Figure 6). Soil urease (approximately 250 μg d^−1^ g^−1^ soil across all treatments) and sucrase (9 mg d^−1^ g^−1^ soil) activities exhibited slight fluctuations across treatments but showed no significant differences, indicating relative stability (Figure 6A,B). In contrast, soil acid phosphatase activity decreased significantly with increasing myo-inositol concentration, reaching its lowest levels at 0.8 and 1.0 g L^−1^, with an approximately 74% reduction compared with the control (Figure 6C). Conversely, soil alkaline phosphatase activity increased markedly at 0.8 and 1.0 g L^−1^, representing an approximately 152% increase relative to the control (Figure 6D).

**Figure 7:**
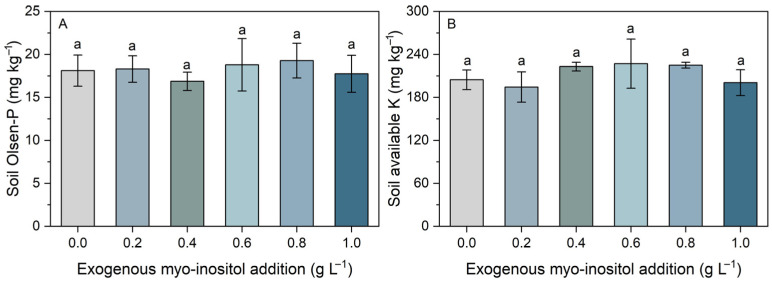
Effects of different concentrations of exogenous myo-inositol (0.0–1.0 g L^−1^) on soil Olsen-P (**A**) and soil available K (**B**) concentrations. Values represent means ± SE (*n* = 4). Different letters above the bars indicate significant differences among treatments (*p* ≤ 0.05).

Soil Olsen-P and available K contents were not significantly affected by myo-inositol application (Figure 7). Overall, soil Olsen-P remained around 18 mg kg^−1^ across all treatments, while soil available potassium was approximately 210 mg kg^−1^. These results indicate that myo-inositol primarily altered phosphatase dynamics without affecting soil P and K availability, suggesting that plant growth responses were driven by physiological or rhizosphere-mediated mechanisms rather than by changes in bulk soil nutrient availability.

### 2.7. Correlations Among Root Morphology, Ion Homeostasis, Antioxidant Capacity, Soil Properties, and Shoot Performance

**Figure 8:**
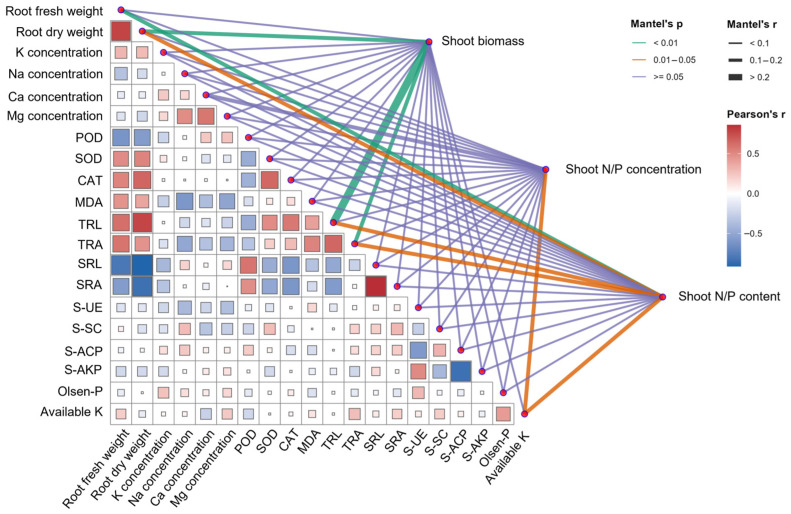
Correlation heatmap and Mantel test network illustrating relationships among root traits, shoot growth, nutrient status, ion homeostasis, antioxidant enzyme activities, and soil enzyme parameters. Pearson’s correlation coefficients (*r*) are shown in the lower triangle, with red and blue squares indicating positive and negative correlations, respectively; color intensity reflects correlation strength. The Mantel test network (right panel) depicts associations between variable groups and shoot biomass, shoot N/P concentration, and shoot N/P content. Line color represents Mantel’s *p* value (*p* < 0.01, 0.01 ≤ *p* < 0.05, *p* ≥ 0.05), and line thickness indicates Mantel’s *r* value. Abbreviations: POD, peroxidase; SOD, superoxide dismutase; CAT, catalase; MDA, malondialdehyde; TRL, total root length; TRA, total root surface area; SRL, specific root length; SRA, specific root surface area; S-UE, soil urease; S-SC, soil sucrase; S-ACP, soil acid phosphatase; S-AKP, soil alkaline phosphatase.

Integrated Pearson correlation and Mantel network analyses revealed coordinated interactions among root morphology, ion homeostasis, antioxidant capacity, soil properties, and shoot performance (Figure 8). Root biomass was strongly positively correlated with total root length (*r* = 0.78) and surface area (*r* = 0.46), but negatively correlated with specific root length (*r* = −0.91) and specific root area (*r* = −0.84). Positive associations were also observed between root biomass and antioxidant enzyme activities (SOD (*r* = 0.52) and CAT (*r* = 0.65)), whereas Na concentration (*r* = −0.35) and POD (*r* = −0.61) were generally negatively correlated with root growth parameters. Mantel test results confirmed significant linkages between shoot biomass and root traits, particularly root dry weight and total root length and area. Shoot N and P contents were significantly associated with root morphology and soil factors, especially soil available K, whereas shoot nutrient concentrations showed weaker relationships (Figure 8). Collectively, these findings demonstrate that enhanced shoot biomass and nutrient accumulation are closely linked to root development, improved antioxidant capacity, and coordinated soil biochemical activity.

## 3. Discussion

### 3.1. Exogenous Myo-Inositol Enhances Growth and Nutrient Uptake in Sugar Beet

The present study demonstrates that exogenous myo-inositol significantly enhanced shoot biomass accumulation in sugar beet, with the greatest effect observed at 0.56 g L^−1^, where shoot fresh and dry weights reached their maximum values (Figure 1 and Figure S2). The dose–response trend, characterized by growth stimulation at moderate concentrations and attenuation at higher levels, aligns with earlier findings that myo-inositol acts as a growth regulator and stress-alleviating metabolite, enhancing photosynthesis and improving cabbage salt stress resistance [27]. Nonetheless, excessive supply may impair metabolic stability, reducing its net physiological benefits [17]. Exogenous myo-inositol has been widely reported to improve plant growth and physiological status under abiotic stresses such as drought, salinity, and heat by stabilizing cellular homeostasis and enhancing antioxidant defense systems [17,19,28,29]. In creeping bentgrass and maize, myo-inositol application increased drought tolerance, promoted antioxidant enzyme activity, and improved growth-related parameters through enhanced redox balance [25,30].

A key feature of the present study is that shoot N and P concentrations remained relatively stable, whereas shoot N and P contents increased significantly at the optimal myo-inositol level (Figure 2). These results suggest that greater nutrient accumulation was largely attributable to biomass expansion rather than increased nutrient concentration on a dry weight basis. The observed divergence between nutrient concentration and content reflects a dilution effect, in which rapid biomass growth spreads nutrients over a greater tissue mass while maintaining relatively stable concentrations [31]. In other words, myo-inositol appears to enhance whole-plant nutrient acquisition and/or internal nutrient use efficiency primarily by stimulating growth and nutrient uptake, rather than by increasing nutrient concentration within tissues. This pattern is also consistent with studies reporting myo-inositol-associated improvements in photosynthetic function, water relations, and overall vigor under stress, which can indirectly increase nutrient uptake by sustaining transpiration streams, root activity, and assimilate supply to roots [30,32].

Notably, soil Olsen-P and available K did not differ significantly among treatments (Figure 7), indicating that the increased shoot N and P contents were unlikely to result from a bulk increase in soil-available nutrient pools. Furthermore, Mantel test results revealed stronger associations between root traits (including root biomass, total root length, and root surface area) and shoot N and P content than with shoot N and P concentration (Figure 8). This pattern supports the interpretation that myo-inositol-driven enhancement of growth and root function promoted nutrient acquisition and allocation at the whole-plant level. Collectively, these findings indicate that moderate exogenous myo-inositol enhances aboveground yield in sugar beet by stimulating biomass accumulation and whole-plant nutrient acquisition, thereby improving growth performance under saline-alkali conditions.

### 3.2. Regulation of Ion Balance and Antioxidant Defense in Sugar Beet by Exogenous Myo-Inositol

Ion homeostasis and oxidative balance are closely interconnected, as ionic stress (particularly excessive Na accumulation) can impair enzyme activity and stimulate the overproduction of ROS [33,34]. The present results indicate that shoot concentrations of essential cations (K, Ca, and Mg) remained relatively stable across treatments, whereas Na levels showed a declining trend in response to myo-inositol application (Figure 3). The preferential reduction in Na without affecting essential cations is physiologically meaningful, since effective Na exclusion or vacuolar compartmentation, together with stable K homeostasis, underpins cellular integrity and salinity tolerance [35,36]. Accumulating evidence indicates that exogenous myo-inositol enhances salt tolerance by improving ionic homeostasis and membrane integrity. For example, in *Chenopodium quinoa* and *Brassica rapa*, myo-inositol treatment mitigated the salinity-induced suppression of essential physiological functions by regulating ionic balance [19,27].

The antioxidant results further suggest that myo-inositol modulated ROS-scavenging strategies rather than uniformly enhancing the activity of all antioxidant enzymes. At moderate myo-inositol levels (0.4–0.6 g L^−1^), SOD and CAT activities were elevated, while POD activity declined compared with the control (Figure 4). This coordinated shift may reflect optimization of the antioxidant network, given that SOD and CAT form the primary enzymatic pathway responsible for detoxifying superoxide radicals through their conversion to H_2_O_2_ and ultimately to H_2_O and O_2_ [37]. Enhanced SOD and CAT activities are frequently reported as hallmarks of myo-inositol-mediated stress mitigation. For example, myo-inositol increased antioxidant enzyme activities and reduced oxidative damage indicators in drought-stressed maize, potentially through the involvement of broader redox regulatory cycles [25]. The observed decrease in POD activity may reflect a redistribution of the antioxidant burden toward CAT-mediated H_2_O_2_ detoxification, or changes in apoplastic peroxidase functions depending on tissue context.

Meanwhile, the stable MDA levels indicate that lipid peroxidation was not significantly altered by myo-inositol application (Figure 4). However, antioxidant activity and oxidative markers are not always inversely correlated, as transient ROS generation may accompany growth and signaling, with enzymatic defenses acting to preserve redox equilibrium [38,39]. Given that myo-inositol serves as a precursor for phosphoinositides and other signaling molecules, its exogenous application may modulate redox signaling pathways and membrane stability [40]. Collectively, exogenous myo-inositol may enhance the adaptive capacity of sugar beet to saline-alkali stress by modulating multiple physiological processes, including coordinated regulation of osmotic balance, antioxidant defense, ionic homeostasis, and photosynthetic system protection. However, the underlying mechanisms remain to be fully elucidated and require further investigation through molecular and physiological approaches.

### 3.3. Exogenous Myo-Inositol Modulates Soil Enzyme Activity and Root Morphology in Sugar Beet

Root morphology results indicate that myo-inositol stimulated overall root system expansion, as evidenced by increased total root length and surface area, with peak values at 0.6 g L^−1^ (Figure 5). In contrast, reductions in specific root length and specific root surface area at intermediate concentrations suggest a shift toward a larger root system composed of relatively thicker or denser roots, rather than merely an increase in fine-root production [41,42]. Such a strategy can be beneficial when plants prioritize transport capacity and structural robustness, potentially supporting sustained nutrient and water delivery to a rapidly growing shoot [43,44]. The strong associations observed between root biomass and morphological traits and shoot biomass highlight the central importance of root expansion as a primary driver of shoot productivity (Figure 8). Myo-inositol metabolism is closely linked to cell wall biosynthesis and cell elongation [45], and disruption of inositol biosynthetic genes impairs root development [46]. Therefore, exogenous myo-inositol may facilitate root structural development, thereby enhancing nutrient uptake capacity and transport efficiency [26].

Soil enzyme dynamics revealed a decrease in acid phosphatase activity and a marked increase in alkaline phosphatase at higher myo-inositol levels, while urease and sucrase activities were not significantly affected by exogenous myo-inositol application (Figure 6). Although soil available P did not change significantly (Figure 7), the shift in phosphatase activity suggests altered soil P transformation processes. Soil enzyme activity is strongly influenced by root exudation and microbial activity [47,48]. Since myo-inositol can act as a signaling molecule and carbon substrate in soil systems [49], its application may influence microbial functional groups involved in P cycling, thereby indirectly affecting plant nutrient acquisition (e.g., increased N and P content; Figure 2). Additional research is necessary to substantiate this possibility.

Collectively, the combined evidence from biomass, nutrient accumulation, ion balance, antioxidant activity, and root morphology support a framework in which moderate myo-inositol application enhances plant performance in saline-alkali soils via coordinated regulation of (i) root development and nutrient capture, (ii) ionic balance, particularly reduced Na^+^ accumulation, and (iii) redox homeostasis (increased SOD/CAT activity). Because soil available nutrients remained unchanged, the growth-promoting effect appears to arise primarily from plant-level physiological and root-functional modulation rather than from enhanced soil nutrient supply. These findings reinforce the emerging view of myo-inositol as an integrative regulator of growth and stress resilience.

## 4. Materials and Methods

### 4.1. Experimental Set-Up

A completely randomized experimental design with six treatments of exogenous myo-inositol (0, 0.2, 0.4, 0.6, 0.8 and 1.0 g L^−1^) was established to evaluate its effects on sugar beet growth and nutrient uptake in saline-alkali soil. The concentration gradient of exogenous myo-inositol was established based on previously published studies [26,27]. The myo-inositol (white powder) was procured from Shandong Zhucheng Haochen Biotechnology Co., Ltd. (Zhucheng, China). Based on previous reports, myo-inositol solutions at the designated concentrations were applied to the root zone of the experimental plants at 23, 32, and 46 days after sowing, with 250 mL applied each time [26]. The soil used in the experiment was collected from the 0–20 cm topsoil layer of cultivated saline-alkali farmland in Wulateqian Banner, Bayannur City, Inner Mongolia (41°08′05″ N, 108°49′60″ E). The soil was air-dried and sieved through a 2 mm mesh prior to use. The soil was air-dried and passed through a 2 mm sieve prior to use. The basic properties of the soil are presented in Table 1. Each pot (20 cm upper diameter, 16 cm lower diameter, and 14 cm height) was filled with 3.0 kg of air-dried soil. All treatments received 3.75 g pot^−1^ of compound fertilizer (N-P_2_O_5_-K_2_O, 12–18-15) to ensure adequate nutrient supply for normal plant growth [50]. Each treatment was replicated four times, resulting in a total of 24 pots. 

### 4.2. Plant Growth Conditions

The pot experiment was conducted in a greenhouse at Inner Mongolia Woxin Agricultural Science and Technology Co., Ltd., located in Wulateqian Banner, Bayannur City, Inner Mongolia, China (41°6′51″ N, 108°59′34″ E). The sugar beet genotype used in this study was IM802. Seeds of uniform weight were surface-sterilized in 30% (*v*/*v*) H_2_O_2_ for 10 min, thoroughly rinsed with deionized water, and imbibed in a saturated CaSO_4_ solution for 12 h. The seeds were then germinated in a dark, humid environment by placing them between two layers of filter paper moistened with deionized water in a growth chamber at 25 °C. Approximately 2 days later, four uniformly germinated seeds were selected and sown in each pot. At 15 days after sowing (two-leaf-one-heart stage), excess seedlings were carefully removed, leaving one healthy plant per pot. All pots were arranged in a completely randomized design and re-randomized weekly throughout the experiment. The greenhouse temperature was maintained at 15–18 °C at night and 25–30 °C during the day. During the growth period, plants were watered daily with deionized water, with the amount adjusted based on weight to maintain soil moisture at 70% of field water capacity, following our previously published method [51], and were cultivated for 60 days (Figure S1).

### 4.3. Shoot Harvest and Measurements

At harvest (60 days after sowing), shoots were cut at the soil surface and weighed to determine fresh biomass. Two newly fully expanded leaves were collected, washed, and immediately frozen in liquid nitrogen for subsequent enzyme activity analysis. The remaining shoot tissues were oven-dried at 105 °C for 30 min, followed by drying at 70 °C for 72 h until constant weight was achieved, and then weighed to determine shoot dry biomass. The dried shoot samples were subsequently ground into fine powder with a stainless-steel multifunctional grinder (DM-200 g, Nanjingdongmai, Nanjing, Jiangsu, China). One subsample (approximately 0.3500 g) was digested with a mixture of concentrated H_2_SO_4_ and H_2_O_2_, and total nitrogen (N) concentration in the shoot tissues was determined using the modified Kjeldahl digestion method [52]. Another subsample (approximately 0.3000 g) was digested with concentrated HNO_3_ and H_2_O_2_ using a microwave-accelerated reaction system (CEM, Matthews, NC, USA) [53]. The concentrations of phosphorus (P), potassium (K), sodium (Na), calcium (Ca), and magnesium (Mg) in the digested solutions were determined by inductively coupled plasma mass spectrometry (ICP-MS; Agilent 7700x, Agilent Technologies, Santa Clara, CA, USA). Shoot nutrient content was calculated as the product of nutrient concentration and shoot dry weight.

For the determination of peroxidase (POD), superoxide dismutase (SOD), and catalase (CAT) activities, 0.1 g of fresh plant tissue was homogenized in 1 mL of extraction buffer in an ice bath. The homogenate was centrifuged at 8000 rpm (9302× *g*) for 10 min at 4 °C, and the supernatant was collected and kept on ice for subsequent analysis. POD activity was determined using the guaiacol method [54], SOD activity was measured by the WST-8 method [55], and CAT activity was assayed using the ammonium molybdate colorimetric method [56]. Reaction mixtures were prepared according to the instructions provided in the commercial assay kits (Nanjing Aoqing Biotechnology Co., Ltd., Nanjing, China). Absorbance changes were measured at 450, 470, 405, and 525 nm, depending on the specific assay [57].

Malondialdehyde (MDA) concentration in leaves was determined using the thiobarbituric acid (TBA) reaction method [58], following the manufacturer’s instructions provided with the commercial assay kit (Nanjing Aokang Biotechnology Co., Ltd., Nanjing, China). Briefly, 0.5 g of fresh leaf tissue was homogenized in 5 mL of 0.1% (*w*/*v*) trichloroacetic acid (TCA). The homogenate was centrifuged at 10,000× *g* for 20 min, and 1 mL of the supernatant was mixed with 2 mL of 0.5% (*w*/*v*) TBA prepared in 20% TCA. The reaction mixture was heated in a boiling water bath for 30 min and then rapidly cooled in an ice bath to terminate the reaction. After centrifugation at 10000× *g* for 5 min, the absorbance of the supernatant was measured at 532 nm and 600 nm.

### 4.4. Root Sampling and Measurements

All visible roots were carefully collected and placed in zip-lock bags. The roots were thoroughly washed with deionized (DI) water to remove adhering soil and stored at −20 °C prior to analysis. Root morphological traits were analyzed by scanning the whole root systems. Root morphological traits were determined by scanning the entire root system. Root samples were spread evenly in a transparent tray (30 cm × 20 cm × 3 cm) filled with a thin layer of water to avoid overlap and scanned at 400 dpi using an EPSON scanner (Expression 1600 Pro, Model EU-35, Seiko Epson Corporation, Matsumoto, Japan). Root images were analyzed using WinRHIZO software (Regent Instruments Inc., Quebec, QC, Canada) to determine total root length and root surface area. After scanning, root samples were oven-dried at 70 °C for 48 h to constant weight, and root dry biomass was recorded. Specific root length and specific root surface area were calculated by dividing total root length or total root surface area by root dry biomass, respectively [59].

### 4.5. Soil Sampling and Measurements

After root sampling, the soil in each pot was thoroughly homogenized, passed through a 2 mm sieve, and collected. One subsample of fresh soil was stored at 4 °C for the determination of soil enzyme activities, while another subsample was air-dried for the analysis of soil available P and available K. The activities of soil urease (S-UE), soil sucrase (S-SC), soil acid phosphatase (S-ACP), and soil alkaline phosphatase (S-AKP) were determined using commercial assay kits: S-UE kit (No. ST5200), S-SC kit (No. G0302W), S-ACP kit (No. ZM4269), and S-AKP kit (No. ST5230) (Nanjing Aoqing Biotechnology Co., Ltd., Nanjing, China). Soil NaHCO_3_-extractable P (Olsen-P) was determined using the sodium bicarbonate extraction-molybdenum-antimony colorimetric method following extraction of air-dried soil with 0.5 mol L^−1^ NaHCO_3_ (pH 8.5). Soil NH_4_Ac-extractable K was extracted with 1 M NH_4_Ac and measured by flame atomic absorption spectrophotometry [60].

### 4.6. Statistical Analyses

A one-way analysis of variance (ANOVA) was performed to evaluate the effects of treatments on shoot, root, and soil traits using SPSS statistical software (SPSS 20.0; IBM SPSS Inc., Chicago, IL, USA). Significant differences among means were determined using Tukey’s honestly significant difference (HSD) test at the 5%, 1%, and 0.1% significance levels (0.01 < *p* ≤ 0.05, 0.001 < *p* ≤ 0.01, and *p* ≤ 0.001). A quadratic regression model was applied to evaluate the responses of shoot fresh weight, shoot dry weight, root fresh weight, and root dry weight to varying concentrations of myo-inositol. Mantel tests were performed using the vegan package (v2.7.1) to examine associations among shoot growth, nutrient status, ionic regulation, antioxidant enzyme activities, root morphology, and soil enzyme characteristics.

## 5. Conclusions

The present study confirms the hypothesis that exogenous myo-inositol reduces the effects of saline-alkali soil stress in sugar beet by enhancing nutrient acquisition, ionic balance, antioxidant capacity, and root development. Biomass responses exhibited a clear concentration-dependent pattern, peaking at 0.6 g L^−1^, with regression analysis indicating an optimum of approximately 0.56 g L^−1^. At this optimal level, shoot and root biomass were maximized, and shoot N and P contents increased significantly despite relatively stable tissue nutrient concentrations, indicating biomass-driven nutrient accumulation. Myo-inositol application improved ionic homeostasis by maintaining stable K, Ca, and Mg levels while reducing Na accumulation at moderate concentrations. Concurrently, antioxidant defense systems were strengthened, as reflected by elevated SOD and CAT activities, suggesting enhanced ROS scavenging and redox balance. Root morphological analysis further revealed significant increases in total root length and surface area, supporting improved nutrient absorption capacity and structural development. Collectively, moderate exogenous myo-inositol functions as a metabolic integrator, coordinating root development, nutrient acquisition, ionic regulation, and antioxidant defense to enhance plant performance under saline-alkali stress. These findings provide theoretical support for the application of myo-inositol as a growth-promoting regulator in sustainable crop production systems; however, additional studies are required to evaluate post-harvest production and quality.

## Figures and Tables

**Table 1:** Basic properties of the soil used in the pot experiment.

pH	EC (ms cm^−1^)	SOC (g kg^−1^)	TN (g kg^−1^)	AP (mg kg^−1^)	AK (mg kg^−1^)
9.25	1.68	5.02	0.24	8.59	157

Notes: pH, 1:2.5, soil:water; EC: electric conductivity, 1:5, soil:water; SOC: soil organic carbon; TN, total nitrogen; AP, NaHCO_3_-extractable phosphorus; AK, NH_4_Ac-extractable potassium.

## Data Availability

Raw data are accessible from the corresponding author upon request. The data are not publicly available due to [data are contained within the article].

## Supplementary Materials

The following supporting information can be downloaded at: https://www.mdpi.com/article/10.3390/plants15071022/s1. Figure S1: Shoot growth performance of sugar beet under different treatments (just before harvest). Figure S2. Responses of shoot fresh weight (A), root fresh weight (B), shoot dry weight (C), and root dry weight (D) in sugar beet to different concentrations of exogenous myo-inositol (0.0–1.0 g L^−1^). Regression analysis was performed using a first-order quadratic equation.

## Abbreviations

The following abbreviations are used in this manuscript:

POD	Peroxidase
SOD	Superoxide dismutase
CAT	Catalase
MDA	Malondialdehyde
TRL	Total root length
TRA	Total root surface area
SRL	Specific root length
SRA	Specific root surface area
S-UE	Soil urease
S-SC	Soil sucrase
S-ACP	Soil acid phosphatase
S-AKP	Soil alkaline phosphatase
ROS	Reactive oxygen species
K	Potassium
Na	Sodium
Ca	Calcium
Mg	Magnesium
N	Nitrogen
P	Phosphorus
