# Supplementary material for: Exogenous Myo-Inositol Promotes Sugar Beet Growth and Nutrient Uptake in Saline-Alkali Soil
Source: Plants (Basel). 2026 Mar 26;15(7):1022. doi: 10.3390/plants15071022 (PMC13074365; doi:10.3390/plants15071022)
Supplement: Supplementary file 1 [file plants-15-01022-s001.zip › plants-4193068-supplementary.pdf]

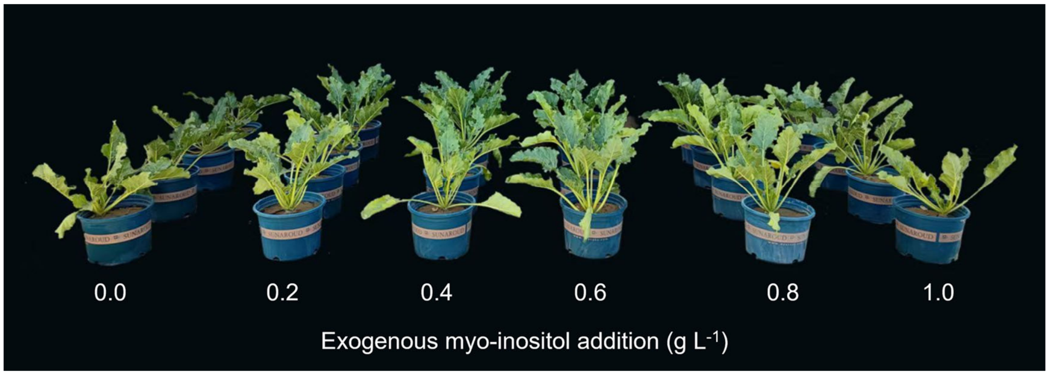

Figure S1. Shoot growth performance of sugar beet under different treatments (just before harvest).

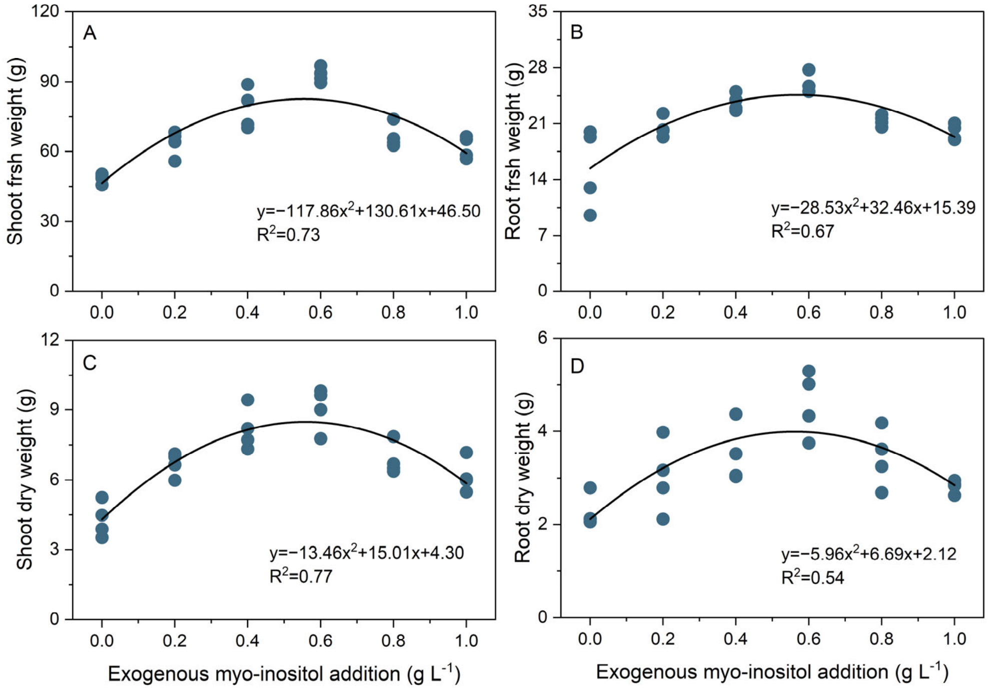

Figure S2. Responses of shoot fresh weight (A), root fresh weight (B), shoot dry weight (C), and root dry weight (D) in sugar beet to different concentrations of exogenous myo-inositol (0.0-1.0 g L^{-1}). Regression analysis was performed using a first-order quadratic equation.
